# Monitoring immunE DysregulAtion foLLowing Immune checkpOint-inhibitioN (MEDALLION): protocol for an observational cancer immunotherapy cohort study

**DOI:** 10.1186/s12885-024-12468-3

**Published:** 2024-06-14

**Authors:** Abigail Gault, Linda Hogarth, Kristian C Williams, Alastair Greystoke, Neil Rajan, Ally Speight, Christopher A Lamb, Alison Bridgewood, Lisa-Jayne Brown-Schofield, Fiona Rayner, John D Isaacs, Jérémie Nsengimana, Christopher J Stewart, Amy E Anderson, Ruth Plummer, Arthur G Pratt

**Affiliations:** 1 Translational and Clinical Research Institute, The Medical School, Newcastle University, William Leech Building, Framlington Place, Newcastle upon Tyne, NE4 2HH UK; 2 Northern Centre for Cancer Care, The Newcastle upon Tyne Hospitals NHS Foundation Trust, Newcastle upon Tyne, UK; 3 Department of Dermatology, Newcastle upon Tyne Hospitals NHS Foundation Trust, Newcastle upon Tyne, UK; 4 Department of Gastroenterology, Newcastle upon Tyne Hospitals NHS Foundation Trust, Newcastle upon Tyne, UK; 5 Department of Rheumatology, The Newcastle upon Tyne Hospitals NHS Foundation Trust, Newcastle upon Tyne, UK; 6 Population Health Sciences Institute, Newcastle University, Newcastle upon Tyne, UK

**Keywords:** Checkpoint inhibitor, Immunotherapy, Cancer, Immune related adverse events, Pathogenesis, Cohort study

## Abstract

**Background:**

Checkpoint inhibitors (CPIs) are widely used in cancer treatment, with transformative impacts on survival. They nonetheless carry a significant risk of toxicity in the form of immune-related adverse events (IrAEs), which may be sustained and life-altering. IrAEs may require high-dose and/or prolonged steroid use and represent a significant healthcare burden. They mimic immune-mediated inflammatory diseases (IMIDs) but understanding of their pathogenesis is limited. The MEDALLION project aims to determine targetable mechanisms of immune dysregulation in IrAE development, employing an immune monitoring approach to determine changes in circulating and tissue resident cells of CPI recipients who do/do not develop them and assessing the contribution of the microbiome in parallel.

**Methods:**

MEDALLION is a non-randomised longitudinal cohort study aiming to recruit 66 cancer patient recipients of anti-PD1/PD-L1, anti-CTLA-4 or combination therapy. Eligible participants include those with malignant melanoma in the adjuvant or metastatic setting, mesothelioma and non-small cell lung carcinoma (NSCLC) treated in the metastatic setting. Comprehensive clinical evaluation is carried out alongside blood, skin swab and stool sampling at the time of CPI initiation (baseline) and during subsequent routine hospital visits on 6 occasions over a 10-month follow-up period. It is conservatively anticipated that one third of enrolled patients will experience a “significant IrAE” (SirAE), defined according to pre-determined criteria specific to the affected tissue/organ system. Those developing such toxicity may optionally undergo a biopsy of affected tissue where appropriate, otherwise being managed according to standard of care. Peripheral blood mononuclear cells will be analysed using multi-parameter flow cytometry to investigate immune subsets, their activation status and cytokine profiles. Stool samples and skin swabs will undergo DNA extraction for 16 S ribosomal RNA (rRNA) sequencing and internal transcribed spacer (ITS) gene sequencing to determine bacterial and fungal microbiome diversity, respectively, including species associated with toxicity. Stored tissue biopsies will be available for in situ and single-cell transcriptomic evaluation. Analysis will focus on the identification of biological predictors and precursors of SirAEs.

**Discussion:**

The pathogenesis of IrAEs will be assessed through the MEDALLION cohort, with the potential to develop tools for their prediction and/or strategies for targeted prevention or treatment.

**Trial Registration:**

The study was registered on 18/09/2023 in the ISRCTN registry (43,419,676).

## Background

Checkpoint inhibitors (CPIs), including anti-programmed cell death protein 1/programmed death-ligand 1 (PD1/PD-L1) and anti-cytotoxic T-lymphocyte-associated protein 4 (CTLA-4) monoclonal antibodies, are now widely used across multiple cancer subtypes in the setting of metastatic, adjuvant and neoadjuvant indications [1]. Indeed, since the US Food and Drug Administration (FDA) approved the first CPI immunotherapy, the realisation of durable responses in subsets of patients across a range of poor-prognosis malignancies has proved transformative. This has come with the unfortunate cost of immune-related adverse events (IrAEs) in many recipients, however. Those of a “severe” nature (grade 3 or above according to common terminology criteria for adverse events, CTCAE) [2], occur in around 55% of people treated with anti-PD1/CTLA-4 combination therapy [3], including colitis (20%), skin rash (10%), hepatitis (15%), endocrine dysfunction (5%), pneumonitis (4%), and inflammatory arthritis (6%); up to 20% of those treated with single agent anti-PD1/PDL1 are similarly affected. Resembling diverse “spontaneous” immune-mediated inflammatory diseases (IMIDs), the toxicities can be life altering. For example, a vitiligo-like depigmenting rash may alter physical appearance permanently, and endocrine dysfunction including hypophysitis or thyroiditis may necessitate lifelong medication and/or disrupt fertility. Some toxicities may be fatal.

Permanent discontinuation of therapy due to toxicity is reported in 5% of trial patients treated with anti-PD1 antibodies [4] and 20% treated with combination therapy, though real-world estimates are as high as 49.9–58.8% and 59.2%, respectively [5]. Most IrAEs occur within 3–14 weeks of CPI treatment, though there are reports of some presenting months to years following cessation of CPI therapy. Neither does a traditional ‘dose-response’ relationship appear to apply, with some patients experiencing severe toxicity after a single dose and others developing none despite years of therapy and profound clinical cancer responses. A link between IrAE incidence and improved cancer response has been demonstrated though positive cancer response is not guaranteed, and IrAEs may complicate assessments of cancer outcomes [6]. Indeed, many IrAEs are managed with early corticosteroid therapy for symptom control, presenting concerns regarding ongoing CPI efficacy, and IrAEs may persist well beyond treatment cessation in some cases.

Despite the use of CPIs for several years out with clinical trials, the pathogenesis of IrAEs remains poorly understood, but evidence for induced dysregulation of systemic adaptive immunity has grown [7]. For example, diversification of the T cell repertoire is a demonstrable consequence of anti-CTLA-4 treatment, with more extensive T cell receptor (TCR) Vβ CDR3 clonotype expansion linked to an increased likelihood of IrAEs in general – potentially mirroring observations in “spontaneous” IMIDs. Moreover, CD8 + rich T cell infiltrates have been observed in tissue from dermatological, gastrointestinal and synovial biopsies from IrAE-affected tissue [8–14]. Finally, anti-CTLA-4/PD-1 combination therapy led to relative expansion and clonal diversification of a normally rare, CD21^lo^ B cell population thought to represent long-lived plasma cell precursors, amongst melanoma patients who developed IrAEs. There is growing evidence of a link between the gastrointestinal microbiome (bacteria colonising the gut) prior to commencement of therapy and the subsequent development of IrAEs, with broader diversity of bacteria more likely to be present in the gastrointestinal tract of patients who respond clinically with improvement of their cancer, and particular bacterial species associated with clinical response or risk of colitis development [15–19].

**Fig. 1:**
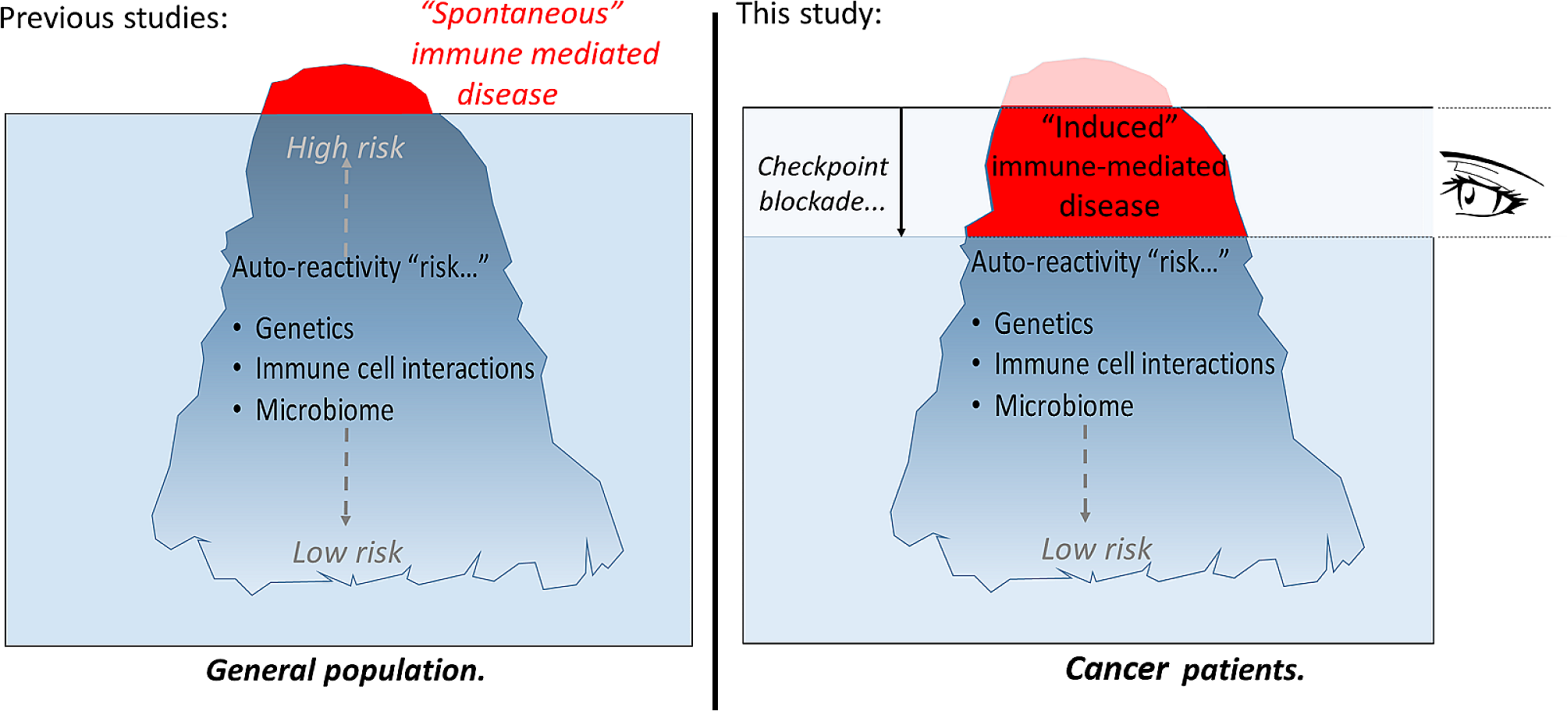
Spontaneous immune mediated disease and MEDALLION study hypothesis. Established immune-mediated diseases are well-studied, and their risk factors are increasingly understood in the general population (left panel). In cancer patients, we hypothesise that checkpoint blockade actively “lowers the threshold” at which immune tolerance is lost, permitting systematic monitoring of the events that trigger clinically manifest disease (right panel). The MEDALLION cohort represents a human “model” of incipient immune dysregulation within which we will test this hypothesis.)

The over-arching hypothesis of the Monitoring immunE DysregulAtion foLLowing Immune checkpOint-inhibitioN (MEDALLION) study is that a continuum of “latent autoreactivity” exists within the general population, and immune and/or microbiome perturbation as a result of CPI therapy lowers the threshold above which a transition to IrAE occurs (Fig. 1). The key biological question we are addressing is how known pathways of immune dysregulation trigger the transition from immune homeostasis to pathology in CPI recipients. In particular, we have demonstrated that interleukin-6 (IL-6) drives signal transduction and activator of transcription-3 (STAT3)-mediated CD4 + T cell activation during the earliest stages of rheumatoid arthritis (RA), a common IMID, and discriminates patients destined to develop RA from those who will develop alternative forms of inflammatory arthritis [20, 21]. “Pre-exposure” of human naïve CD4 + T cells to levels of IL-6 circulating in early RA enhances both their proliferation and activation following subsequent TCR stimulation, suggesting a mechanism by which immune dysregulation may be promoted in early disease [22]. In addition, STAT3 pathway activation has been associated with other IMIDs, such as inflammatory bowel disease [23, 24] and experimental autoimmune encephalomyelitis, an animal model of multiple sclerosis [25]. Whether similar mechanism(s) underlie IrAE development remains to be explored – acknowledging a diversity of plausible alternatives.

## Methods

The current version of the study protocol and associated documentation is available as an Online Supplementary File.

### Study objectives

The study’s primary objective is to establish a clinically well-characterised and immune-phenotyped inception cohort of patients with cancer commenced on immune CPI therapy, thereby enabling the study of immune dysregulation that precedes IrAE development. In order to interrogate mechanisms of IrAE development we will focus on a range of biological measurements at baseline and during therapy, forming the basis of secondary and exploratory objectives. These will include the frequency and activation status of peripheral blood mononuclear cell (PBMC) subsets determined using multi-parameter flow cytometry, the gut and skin microbiome composition, serum cytokine mediators and, where possible, cellular composition of affected tissue.

The secondary objective of the study is to determine whether STAT3 phosphorylation (pSTAT3) in circulating CD4 + T cells of CPI recipients predicts IrAE development at baseline and/or following initiation of treatment.

The exploratory objectives of the study are:

(i) to determine whether baseline and/or dynamic changes in microbiome composition in the skin or gut precede IrAE development in CPI recipients.
(ii) to identify immune cellular phenotypes whose dynamic frequency amongst CPI recipients predicts IrAE development.
(iii) amongst CPI recipients who develop clinically significant colitis and/or skin dermatoses as adverse reaction(s) to treatment, to characterise immune infiltrates of lesional *versus* non-lesional tissue.
(iv) to determine whether circulating cytokine profiles of CPI recipients predicts IrAE development at baseline and/or following initiation of treatment.

### Study design

MEDALLION is a single centre, prospective, longitudinal observational cohort study undertaken from the Northern Centre for Cancer Care, Newcastle upon Tyne, UK. All enrolled participants will receive treatment with combination or single agent CPI therapy routinely as standard care, remaining under study follow up for up to 10 months following CPI commencement or until they develop a “significant IrAE” (SirAE) defined according to pre-determined criteria specific to the affected tissue/organ system - whichever is sooner. In keeping with European Society for Medical Oncology (ESMO) practice guidelines, MEDALLION study-specific SirAE definitions generally align with CTCAE Grade 3, but are modified to reflect the significant impact of certain grade 2 IrAEs on quality of life, for example where warranting treatment with corticosteroids; they are summarised in Table 1.

**Table 1:** Grades 2 and 3 IrAE definitions align with ESMO criteria [3]

Organ system(s)	MEDALLION definition of SirAE
Skin	Any grade 3 IrAE or above *or* CTCAE grade *≥* 2 IrAE, warranting topical/systemic steroids and/or (in guidance with consulting dermatologist) warranting diagnostic skin biopsy. *or* Physician-diagnosed new onset vitiligo
Gastrointestinal tract	Any grade 3 IrAE or above *or* Diarrhoea, CTCAE grade *≥* 2 persisting *≥* 3 days, warranting steroid treatment and/or endoscopically/histologically confirmed colitis. (Severity grade 2 is 4–6 liquid stools per day over baseline, or abdominal pain or nausea or nocturnal episodes) *or* CTCAE grade *≥* 2 hepatitis warranting steroids
Endocrine	Any grade 3 IrAE or above *or* Symptomatic autoimmune thyroid disease confirmed biochemically *or* Hypophysitis confirmed biochemically and/or radiologically
Musculoskeletal	Any grade 3 IrAE or above *or* Objectively observed inflammatory arthritis
All other	Any grade 3 IrAE or above

### Study population

**Fig. 2:**
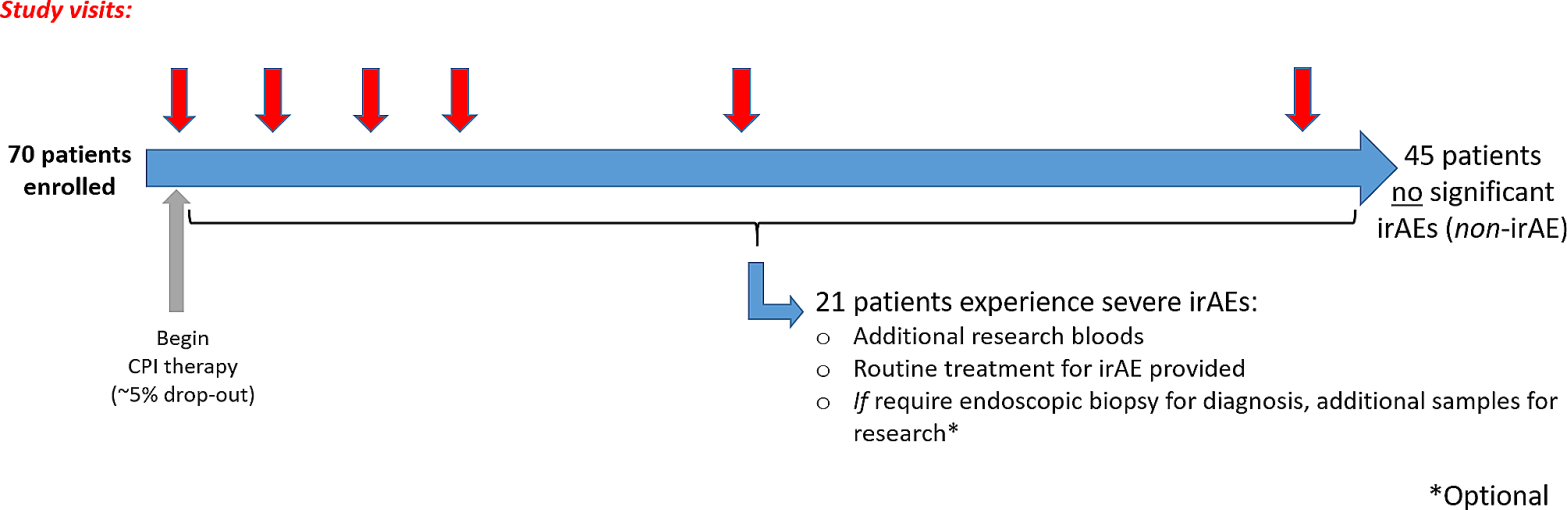
MEDALLION enrolment plan and design. Clinical assessments will take place at all patient visits, planned to coincide with routine hospital visits, and red arrows indicate time points at which 53 ml research bloods will be drawn (providing irAE has not occurred); additional research bloods +/- biopsy will be obtained at the time of incident SirAE)

The MEDALLION study aims to recruit approximately 70 patients treated with CPIs. Accounting for withdrawals this should provide approximately 66 patients completing the study as shown in Fig. 2. The literature suggests that ~ 50% of patients on combination, and ~ 15% of those on single-agent immunotherapy will experience IrAEs of CTCAE *≥* grade 3 severity, and we anticipate a conservative 1:2 ratio of combination: single-agent recipients in our cohort. Hence, incorporating a 5% drop-out rate, approximately 21/66 patients in our study will experience SirAEs.

## Eligibility criteria

### Inclusion criteria

1. Male or female patient *≥* 18 years of age.
2. Confirmed diagnosis of malignant melanoma, NSCLC or mesothelioma.
3. Shared decision by oncologist and patient to proceed with CPI treatment, either with the combination of ipilimumab and nivolumab, or with single-agent nivolumab, pembrolizumab or atezolizumab as standard of care.
4. Patient is judged as being capable of understanding the information sheet and of giving informed consent according to the Mental Capacity Act 2005.
5. Written informed consent to participate in the study.

### Exclusion criteria

1. Known pre-existing autoimmune or immune-mediated inflammatory disease requiring immunomodulatory treatment, including (but not limited to) inflammatory bowel disease (Crohn’s disease, ulcerative colitis) autoimmune endocrinopathy or hepatitis, vitiligo and inflammatory arthritis.
2. Received enteral or parenteral steroids within past month (topical, inhaled or intranasal permitted).
3. Previous treatment with CPI therapy.
4. Vaccination within the past 4 weeks, except COVID-19 vaccination permitted.
5. Known chronic infection.
6. Current pregnancy, or pregnancy planned within next 6 months.
7. Inability to provide informed consent and/or undergo any of the procedures mandated by the study.

### Screening visit

Before undergoing any study related procedure (including screening procedures), all potential participants will provide full, written informed consent. Participant demographics, full medical history including cancer history, general physical examination, and patient symptom questionnaires are completed at the screening visit. Standard of care blood tests, research blood tests and six skin swabs (Table 2) are taken, and a pregnancy test is taken for women of childbearing potential. Sites undergoing skin swab were: forehead, upper chest, upper back, dorsum of the hand and forearm; vitiligo-like depigmenting rashes were also swabbed if present. The full schedule of events is shown below in Table 2. Participants at consent can decide to contribute optional additional samples including: regular stool samples for microbiome/mycobiome analysis, accompanied by optional completion of a nutritional questionnaire, and optional gastrointestinal or dermatological tissue if they develop a SirAE affecting these sites that trigger the need for biopsy as part of routine care.

**Table 2:** Schedule of events

VISITS	VISIT 1^a^	VISIT 2^b^	VISIT 3^b^	VISIT 4^b^	VISIT 5^b^	VISIT 6^b^	END OF STUDY VISIT^C^	Unsched-uled Visit^d^	SirAE Visit^e^	Optional ‘special case’ SirAE biopsies^f^
**TREATMENT CYCLE (Combination ipilimumab/nivolumab, single agent pembrolizumab, nivulumab or atezolizumab, or switch between these therapies or to another CPI treatment / regimen**	**CYCLE 1** **-14 days** **(Baseline)**	**CYCLE 2 +/-7 days**	**CYCLE 3 +/-7 days**	**CYCLE 4 +/-7 days**	**CYCLE** **dependent upon drug regimen** ^**b**^ **+/-7 days**	**CYCLE** dependentupon drug regimen^**b**^ **+/-7 days**				
**Procedures**										
Discuss Study	X									
Sign consent form	X^g^								X^h^	X^h^
Pregnancy test	X	X^i^	X^i^	X^i^	X^i^	X^i^	X^i^	X^i^	X^i^	X^i^
Concomitant meds recording	X	X	X	X	X	X	X	X		X
General physical examination	X	X^j^	X^j^	X^j^	X^j^	X^j^	X^j^	X^j^		X^j^
Adverse event assessment	X	X	X	X	X	X	X	X		X
Questionnaire^k^	X	X	X	X	X	X	X	X		X
Blood test (NHS Lab)^l^	X	X	X	X	X	X	X	X		X
Research blood test^m^	X	X	X	X	X	X	X		X	X
Skin swabs^n^	X	X	X	X	X		X		X	
Stool Sample (optional)^o^	X	X	X	X	X		X		X	
Photography of skin IrAEs (optional)^p^		X	X	X	X	X	X	X		
Skin Biopsy for research (optional)^q^									X	X
Endoscopic biopsies for research (consenting patients with GI SirAEs only)^r^.									X	X

a Baseline procedures should be performed within 14 days of receiving of administration of the first dose of combination ipilimumab/nivolumab, or single agent pembrolizumab, nivolumab or atezolimumab

b Study visits to occur within +/-7 days of receiving CPI for visits 2, 3, 4, 5 and 6. Cycles of CPI treatment are scheduled to occur at:

i Nivolumab (3-weekly)/ipilimumab (3- or -6 weekly) x 4 cycles, followed 6 weeks after combination treatment by 4-weekly single agent Nivolumab) (melanoma) weeks 3, 6, 9, 15 and 39 (i.e. cycles 2, 3, 4, 5 and 11)

ii Nivolumab (melanoma) weeks 4, 8, 12, 16 and 40 (i.e. cycles 2, 3, 4, 5 and 11)

iii Pembrolizumab (3-weekly, followed by 6-weekly after week 9 or 12) (melanoma), weeks 3, 6 and 9, then week 15 or 18 and week or 39 or 36 (i.e. cycles 2, 3, 4, 5 or 6 and 9)

iv. Pembrolizumab (6-weekly) (melanoma) weeks 6, 12, 18, 24 and 36 (i.e. cycles 2, 3, 4, 5 and 7)

v Pembrolizumab 4 × 3-weekly followed by 6-weekly after week 12 (NSCLC), weeks 3, 6, 9, 18 and 36 (i.e. cycles 2, 3, 4, 6 and 9)

vi Atezolizumab 4-weekly (NSCLC) weeks 4, 8, 12, 16 and 40) (i.e. cycles 2, 3, 4, 5 and 11)

vii Nivolumab(3-weekly)/ipilimumab(6-weekly) (Mesothelioma) weeks 3, 6, 9, 15 and 36 (i.e. cycles 2, 3, 4, 6 and 13)

c End of Study Visit, if applicable, i.e. if a patient’s treatment ends before completing the follow up visits and for a reason other than a SirAE Visit to be completed within 14 days of the decision to end treatment

d An unscheduled visit is only required at the discretion of the investigator if the participant contacts the research team with a new possible irAE (see *Sect. 7.6*)

e Wherever possible the SirAE visit (and all of the listed procedures except optional gut biopsy) should be combined with the scheduled or unscheduled visit at which the SirAE is determined to have occurred, unless a scheduled or unscheduled visit has already been performed within post 7 days (or 14 days if a standard of care gut or skin biopsy with a research biopsy is being performed) of identification of the SirAE (see Sect. 7.7), in which instance the SirAE procedures should be carried out alone. Some of the SirAE procedures may require a separate visit when the SirAE event can only be confirmed when blood results are available or for gastrointestinal or skin SirAEs where the patient consents to gut or skin biopsy

f Optional ‘special case’ SirAE biopsies when standard of care gut or skin biopsy occur later than 14 days post initial identification of an SirAE whilst on CPI treatment. Optional research biopsies will be allowed at any time point that standard of care biopsies occur, until what would have been the patient’s final scheduled study visit if no SirAEs had occurred. Research procedures listed in the above table will be performed on the same day as the research biopsy (if not already performed within 7 days of the biopsy)

g Main study and (optional) for stool sample donation and also (optional) for a skin biopsy where indicated

h For obtaining gut biopsies for research from patients experiencing gastrointestinal SirAEs undergoing lower gastrointestinal tract endoscopies as part of routine care under direction of consulting gastroenterologist (optional), or for a skin biopsy where indicated (optional)

i Female patients will be asked whether they might be pregnant at each study visit and a pregnancy test conducted if relevant

j Physical exam as applicable, as per standard of care

k Symptom-directed questionnaire; Appendix 1

l FBC, U&E, LFT, TFT, Magnesium (standard of care tests) ESR, CRP (non-standard of care tests). At an End of Study visit FBC to be done as a research blood, if not being done as standard of care

m 4 × 10mL EDTA, 1 × 8.5mL serum, 1 × 10mL heparin

n. Skin swabs from forehead, upper chest, upper back, dorsum of the hand and forearm at each visit with potential additional swab from a site of vitiligo-like depigmenting rash if occurs. ***Skin swabs applicable only for up to the first 53 evaluable participants***

o Only for patients who consented at baseline to stool sample collection. ***Stool collection applicable only for up to the first 53 evaluable participants***

p Only for patients who consented at baseline to photography of skin IrAEs

q Only for patients who consent to optional skin biopsy of affected skin

r Only for patients who consent at time incident IrAE determined

### Follow-up: post-cycle 1,2,3,4, and 9 months

Patients undergo formal study clinical review post-cycles 1, 2, 3, and 4 months of CPI treatment and at 9–10 months depending on the CPI regimen as detailed in Table 2 to coincide with routine care visits to hospital. If patients change CPI therapy prior to completing the scheduled 6 study visits, then they can continue within the study from cycle 1 of their new treatment until they have completed 6 scheduled study visits in total. Adverse events and concurrent medications are recorded at each visit in addition to clinical review. Clinical review will include collation of a detailed symptom directed questionnaire from patients, general physical examination, collection of skin swabs and routine blood tests. Participants who have consented to optional stool samples will also provide these following each visit, they will furthermore be asked to submit a completed dietary questionnaire.

### Adverse events and principle clinical end point

The reporting of adverse events will be undertaken at each visit, with these graded as mild, moderate, or severe, and allocation of a numerical grade according to the CTCAE version 5^2^. AEs are classified in terms of their relatedness to CPI therapy as follows: unrelated, unlikely, possible, probable, definitely and not assessable.

*Significant* IrAEs for purposes of the MEDALLION study (SirAEs) are defined according to strict criteria and recorded at scheduled/unscheduled visits, being those considered “definitely” or “probably” related to administration of the CPI therapy, and meeting organ system-specific criteria as outlined in Table 1. SirAEs constitute the principle clinical endpoint of the study.

Where a definite IrAE is determined to have occurred by the investigator but the above SirAE criteria are not fulfilled this will be recorded as a “non-significant IrAE” and the patient will remain in follow-up.

### IrAE ‘ad hoc’ visit

Participants can directly request an unscheduled visit if they suspect they are developing an IrAE, or they may be identified at routine clinical review. Research specific biological sampling will take place at the time of incident IrAE.

### Withdrawal criteria

Participants can decide to withdraw their consent at any point during the study. The clinical investigator may withdraw a participant from the trial at any time if this is considered necessary, and for any reason including:

i. Symptomatic deterioration.
ii. Participant withdrawal of consent or inability (through incapacity or otherwise) to provide consent for study-specific procedures to proceed.
iii. Significant protocol deviation or non-compliance, including failure to attend for > 2 consecutive visits.
iv. An adverse event such that continuation of CPI therapy is no longer appropriate, even if SirAE criteria are not fulfilled.
v. Termination of the clinical trial by the sponsor.
vi. Investigator’s discretion that it is in the best interest of the participant to withdraw.
vii. The patient has fulfilled the SirAE criteria and completed their IrAE ad hoc visit.

### Statistical considerations

The primary objective of the study is to generate pilot data from a substantive cohort to support hypotheses that may be tested and/or validated in future investigations. Statistical considerations are applied to address secondary (hypothesis testing) and exploratory (hypothesis generating) objectives.

#### Sample size justification

Observations made in a separate study, BIOFLARE [26], on pSTAT3 expression in a subset of circulating CD4 + T cells of RA patients in remission who develop disease flare following treatment cessation *versus* those who do not, were used as a basis for sample size calculation. Assuming CPI recipients with and without SirAEs will display a difference of similar magnitude in CD4 + T cell pSTAT3, enrolment of 66 participants with complete datasets will afford MEDALLION 80% power to detect a difference of ~ 24% between groups at alpha 0.05 (*pilot data used currently in preparation for publication; personal communication, JDI and F Rayner*). However, the longitudinal nature of our study is an important aspect, as it will allow taking account of the exact time the IrAEs occur. There are very few relevant similar datasets but one recent report found in a Swiss population three immune-related predictors of IrAEs in melanoma patients treated with CPIs: CXCL10, IL-10 and regulatory T cell (Treg) levels with hazards ratios of 12.6, 4.0 and 3.4, respecitvely [27]. Detecting effect sizes broadly similar to these with power 80% at significance level 0.05 requires a minimum of 21 events. Based on the anticipated 32% participants experiencing SirAEs, recruitment and following up of the aforementioned 66 patients will be sufficient for such analyses.

#### Analysis plan

A broad range of measurable immune parameters will be evaluated in MEDALLION, in addition to the microbiome, but the aim of the main statistical analysis (secondary study objective) is to addresses the hypothesis that pSTAT3 expression in circulating CD4 + T cells pSTAT3 predicts SirAE development. The study design ensures that, in addition to testing a specific hypothesis, our unique cohort will form a substrate for a wide range of exploratory analyses in relation to cellular immunity and the microbiome during the development of SirAEs. Descriptive statistics will be used to describe recruitment rates, reasons for refusal to participate, refusal rates for optional procedures and missing data. They will also be applied to compare baseline circulating CD4 + T cell pSTAT3 between patients developing a significant SirAE *versus* those that do not (e.g. Mann-Whitney U test), with similar comparisons made up to the time of incident SirAE or matched time point. Exploratory analyses in relation to other biological parameters will then be undertaken in a similar manner. Markers’ association with time-to-SirAE will furthermore be evaluated using Cox proportional hazards regression. Significantly predictive markers will be graphically displayed by Kaplan-Meier curves after dichotomisation. Mixed effect logistic regression, a form of general linear mixed model (GLMM), will be applied to repeated measurements to test association between SirAE occurrence and marker longitudinal trends. Statistically significant trends will be depicted graphically. GLMM framework is flexible and will allow to exploratively adjust key clinical covariates although their number will be limited by available sample size.

### Data handling

The number of patients approached, interested in taking part and screened will be collected via a log completed by staff conducting screening. Data for an individual patient will be collected by the study PI or their delegated person and recorded in the secure, password-protected electronic case report form (eCRF) for the trial. Patient identification on the eCRF will be through a unique trial identifier number. A record linking the patient’s name to the unique identifier number will be held securely at the trial site, and is the responsibility of the PI. As such, patients cannot be identified from eCRFs.

The PI or delegated person will monitor completeness and quality of data recording in eCRFs and will correspond regularly with investigators to avoid missing data where possible and ensuring continuous high quality of data. Patients will complete the paper assessment tools as required. The tools will also only be identified using the unique patient identifier number. Overall responsibility for data collection lies with the PI. Data collected on paper assessment tools will be entered onto a secure validated clinical data management system. A study identifier number will be used to identify participants on all paper data collection forms throughout the duration of the trial. Data will be handled, computerised, and stored in accordance with the Data Protection Act 2018. No participant identifiable data will leave the study site. The quality and retention of study data will be the responsibility of the PI. All study data will be retained in accordance with the latest Directive on Good Clinical Practice (2005/28/EC) and local policy.

Staff involved in the conduct of the trial, including the PI and study staff involved in screening and intervention will have access to the site files for patients at the hospital study site. Clinical information shall not be released without the written permission of the participant, except as necessary for monitoring and auditing by the Sponsor or regulatory authorities. Secure pseudonymised electronic data may however be released to named members of the study team for analysis purposes. The PI and trial site staff involved with this trial may not disclose or use for any purpose other than performance of the trial, any data, record, or other unpublished, confidential information disclosed to those individuals for the purpose of the trial.

All trial data will be stored securely in accordance with Good Clinical Practice (GCP) and the Sponsor guidelines (Newcastle JRO standard operating procedures; SOPs). Any personal identifiable information will be stored at the study site or Newcastle upon Tyne Hospital Foundation Trust (NuTH) archiving facilities, for up to 5 years before secure disposal.

## Discussion

Developing understanding of the pathogenesis of IrAEs is fundamentally important to attempt to predict and effectively target toxicity within the CPI-treated population. The mechanistic insights this could provide for other IMIDs is also important, due to probable shared disease pathways and difficulty examining the early pre-symptomatic phase of IMIDs.

The design of the MEDALLION study allows for close monitoring during the period in which IrAEs are most likely to arise, without creating any additional visit burden for patients, which was well received by our patient & public involvement group. Within the study, clinical progress over time including; CRP, ESR, neutrophil & lymphocyte counts are captured alongside thyroid, haematological, renal and liver function to identify any subclinical biochemical toxicity. During each participant visit, blood taken for research is processed according to the study SOPs and stored and tracked using a laboratory inventory management system (LIMS).

Future planned analysis of this blood will allow multiple questions to be explored. Deep immune-phenotyping will be undertaken via flow cytometric analysis from whole blood and PBMCs, including investigation of cytokine levels. Stool samples and skin swabs will undergo 16 S rRNA sequencing to determine bacterial and fungal microbiome composition, allowing for comparison between the group developing IrAE and those without. In downstream work, additional sampling in MEDALLION will permit genotyping and epigenetic profiling of residual PBMCs and/or whole blood as well as histological and molecular characterisation of gastrointestinal or dermatological tissue samples collected at SirAE timepoints, with the potential for single cell sequencing of these specimens.

Whilst other groups nationally and internationally are developing biobanks of samples from patients treated with CPIs, our study methodology allows for serial sampling from baseline, and includes patients treated with CPIs who do not develop IrAEs which act as a robust comparator for those who develop IrAEs. MEDALLION will provide a valuable experimental medicine resource in its own right, in addition to acting as a reference for the design of future studies investigating IrAE pathogenesis, and as a source of pilot data for larger collaborative projects now and in the future. Our skin swab protocol serially samples multiple sites, including those most likely to be affected by dermatological IrAEs to enable a thorough assessment of dermatological dysbiosis at affected sites which hasn’t been explored in similar published cohorts to date. Sampling fungal mycobiome from stool samples and skin swabs has also not previously been explored in CPI-treated patients on this scale.

MEDALLION has been implemented as a single centre study and its findings cannot, therefore, necessarily be assumed to be applicable across geographically and demographically diverse populations; validation of key findings will be required on the path to translating them for the benefit of all patients. That said, CPI treatment regimens applied as part of usual care adhere to nationally agreed guidelines (ensuring a degree of generalisability), and the design facilitates consistency of biological sample handling. It is furthermore acknowledged that any effort to dichotomise the severity of diverse irAEs on clinical grounds carries an unavoidable “arbitrary” element. Rather than adhere to a single, fixed CTCAE grade 3 cut-off across *all* types of irAE, however, and with the goal of predicting and describing the immunopathology of those of the most impactful for patients, we included certain grade 2 irAEs where intervention with steroid or hormonal therapy was indicated or where significant inflammatory disease (including arthritis) ensued. This decision was informed by discussions with patients during protocol development.

To summarize, this study will provide a greater understanding of changes within the microbiome, circulating immune cells and tissue-resident changes during IrAEs in patients treated with CPIs, with the aim of improving knowledge about their pathophysiology that may, in turn, inform understanding of the biology underpinning other IMIDs. Such work should contribute to efforts to improve care for people suffering with immune-mediated disease be it spontaneous or drug-induced, through the development of predictive tools and/or strategies for targeted prevention or treatment.

## Data Availability

All of the data pertinent to this manuscript is available within it and the associated supplementary information.

## Abbreviations

CPIs: Checkpoint inhibitors
CTCAE: Common Terminology Criteria for Adverse Events
CTLA-4: Cytotoxic T lymphocyte-associated protein 4
eCRF: Electronic case report form
GCP: Good clinical practice
GLMM: Generalised linear mixed model
IrAEs: Immune related adverse events
IMIDs: Immune mediated inflammatory diseases
LIMS: Laboratory information management system
NSCLC: Non-small cell lung cancer
PBMCs: Peripheral blood mononuclear cells
pSTAT3: Phosphorylated STAT3
NuTH: Newcastle upon Tyne Hospital Foundation Trust (NuTH)
PD-1: Programmed cell death protein 1
PD-L1: Programmed death-ligand 1
RA: Rheumatoid arthritis
SirAE: Significant immune related adverse event
SOPs: Standard operation procedures
Treg: Regulatory T cell
